# Metagenomic search strategies for interactions among plants and multiple microbes

**DOI:** 10.3389/fpls.2014.00268

**Published:** 2014-06-11

**Authors:** Ulrich Melcher, Ruchi Verma, William L. Schneider

**Affiliations:** ^1^Department of Biochemistry and Molecular Biology, Oklahoma State UniversityStillwater, OK, USA; ^2^Foreign Disease-Weed Science Research Unit, United States Department of Agriculture – Agricultural Research ServiceFort Detrick, MD, USA

**Keywords:** e-probes, BLAST, microbial consortia, taxonomic classification, databases, EDNA

## Abstract

Plants harbor multiple microbes. Metagenomics can facilitate understanding of the significance, for the plant, of the microbes, and of the interactions among them. However, current approaches to metagenomic analysis of plants are computationally time consuming. Efforts to speed the discovery process include improvement of computational speed, condensing the sequencing reads into smaller datasets before BLAST searches, simplifying the target database of BLAST searches, and flipping the roles of metagenomic and reference datasets. The latter is exemplified by the e-probe diagnostic nucleic acid analysis approach originally devised for improving analysis during plant quarantine.

## BACKGROUND

A microbe entering a plant whether transmitted by a vector, through abrasion, or by wind-driven rain encounters an environment, the phytobiome, which consists of the plant and all microbes associated with it. Much has been learned about how an individual microbe interacts with a more-or-less pristine plant (Baker et al., 1997). Yet, investigations of microbes associated with plants often reveal the presence of multiple microbes. Multiple infections with multiple viruses are increasingly being discovered (Al Rwahnih et al., 2009; Villamor and Eastwell, 2013). Multiple species of bacteria are often found in endophytic association with plants (Ding et al., 2013; Ma et al., 2013). These same virus- or bacteria-infected plants may also harbor fungi or oomycetes. Interactions between phytobiome microbes have consequences for the plant. Microbe infection often induces systemic acquired resistance (Rojas et al., 2014), an alteration of the physiological status within the plant which alters the outcomes of arrival of other microbes. Virus infection by one virus can exacerbate disease symptoms in some cases, synergistic viral disease, or reduce the effects of introduction of a second-virus, cross-protection (Palukaitis, 2011) in others. Several microbes are known to be biocontrol agents that control the proliferation of other microbes associated with plants (Santoyo et al., 2012).

Increasingly, investigators want to consider all components of the phytobiome in their analyses. To this end, we consider here approaches based on next generation sequencing (NGS) to detect microbial components of the phytobiome. NGS has enabled large-scale metagenomics, which is a gene-based study of all organisms associated with a particular sample (Rucker et al., 2013; Wang et al., 2013). Time-efficient and -effective means of examining NGS databases to identify organisms that contribute to the metagenome are needed to study multiorganism consortia. Which organisms are associated with one another? Which organisms exclude each other?

These questions fuel a need for the taxonomic classification of NGS DNA or RNA sequence reads. Such classification is important also for various other fields of study including ecology, diagnostics, and homeland security (Macdiarmid et al., 2013). The responses of marine ecosystems to climate change and anthropogenic pollution may be revealed by studies of the changing diversities of marine microbes (Coelho et al., 2013). Understanding the importance of the presence of certain microbial species in the human microbiome fuels attempts to adjust diets to achieve the most beneficial balance of bacterial species (Cox et al., 2013; Umu et al., 2013). Taxonomic profiles of microbes are important in understanding complex human diseases, such as inflammatory bowel disease, type 2 diabetes, and obesity (Segata et al., 2012; Cani, 2013). The balances of rhizosphere microbes among phytopathogenic bacteria, plant-growth-promoting bacteria, and, bacteria that can be pathogenic to animals and humans need further investigation (Mendes et al., 2013).

In diagnostic analysis, taxonomic classification of sequence reads is particularly important in the case of diseases whose etiology is unknown or whose symptoms could be produced by multiple species of infectious agents (Bernardo et al., 2013). Novel plant viruses have been identified by metagenomic means (Al Rwahnih et al., 2009; Roy et al., 2013a). The exploration of microbes associated with archaeological remains promises to enlighten the discussion of the emergence of infectious diseases in historic and prehistoric times (Gibbons, 2013; Smith et al., 2014).

Microbe–plant interaction studies should consider third (or higher order) partners when studying binary interactions of microbes with plants. The first step in such consideration is the now traditional metagenomic survey of the organismal consortium including all microbes present in representative samples. This is followed by use of the sequence reads as queries of general databases, a time-consuming process. For ecological purposes, once particularly interesting organisms and their interrelations have been targeted, investigators may concentrate on the fluctuations of population sizes of particular taxa, simplifying the search, as described below.

## TAXONOMIC CLASSIFICATION OF NGS READS: APPROACHES

### OVERVIEW

With the advent of rapid, accurate, and less-expensive nucleic acid sequencing technology, phenomenal amounts of sequence data are being generated. The lengths of reads and the kinds and quantities of sequencing errors are characteristic of the sequencing methods (Dröge and McHardy, 2012). The rate of production for sequences is currently highest for Illumina technology, which can average 3.1 × 10^9^ nt/h. The ability to analyze NGS data is growing also but at a much slower pace (Hunter et al., 2012), creating an analysis bottleneck in achieving many of the goals of metagenomic studies (Dröge and McHardy, 2012).

Taxonomic classification of NGS reads inherently consists of comparison of two datasets, the NGS reads, and a compilation of sequences of known taxonomic origin. The latter is frequently the non-redundant version of GenBank/EMBL/DDBJ nucleotide databases. The comparison is done typically using the BLASTn algorithm with the NGS reads as queries and the nr/nt database as target for the searches. Currently, the most typical analytical method for metagenomic data is to use sequence reads as queries of the general nucleotide databases to find the best matches to each query, followed by a taxonomic assignment of the read to an organism using software, such as MEGAN, Darkhorse, or Kirsten (Teeling and Glockner, 2012).

Four approaches to closing the gap between the generation of sequence reads and their analysis are being pursued: further improvement in computational speed; condensing the NGS reads dataset; simplifying the known sequence dataset; and flipping the roles of the two datasets. These are discussed below.

### COMPUTATIONAL SPEED

Computational speed can be enhanced by using multiple compute nodes. However, facilities offering massively parallel computing are often not available at the location of the sequence generation unit. Thus, reads need to be transported to the computing unit either using large bandwidth communications or physically, by sending high-capacity hard drives. In addition, speed can be increased by breaking the total pool of reads into multiple subpools. The fragmentation may remove overlap possibilities, a process that could lead assembly into a non-justified sequence recombination. Considerable acceleration of taxonomic assignment at the generic level (and at the species level with lower sensitivity) can be obtained by restricting searches to finding only complete matches to k-mer words, as implemented in Kraken (Wood and Salzberg, 2014).

### CONDENSING THE NGS READS DATASET

The sequences can first be subjected to an assembly process and the resulting contigs can be queries in BLASTn searches. Assemblers such as Genovo, MetaIDBA, MetaVelvet, and MAP are used, but do themselves take considerable time to finish the assemblies of large datasets (Pell et al., 2012). Since fewer, but longer, sequences are used, such searches may be faster than searching with the raw data. However, the time required in assembly and the hazards of misassembly may negate the advantage. In addition, most assembly methods require a filtering of the read data to remove low-abundance reads which may come from minor community components. Recently, the use of graph theory on short k-mers using a Bloom filter was proposed (Pell et al., 2012) and reduced the memory requirements for large assemblies of metagenomic data and did not require the discarding of reads.

Another approach to simplifying sequence datasets is the use of bioinformatic or molecular approaches in pre-sequencing or post-sequencing steps that enrich for pathogen- or microbe-related sequences (Melcher et al., 2008). For example, multiple researchers have utilized the pool of small RNAs (sometimes called the degradome) as a target pool of nucleic acids that are enriched for viral sequences via plant defense responses (Donaire et al., 2009; Kreuze et al., 2009; Pantaleo et al., 2010; Kashif et al., 2012; Li et al., 2012; Loconsole et al., 2012; Roy et al., 2013a,b). Roy et al. (2013a,b) added subtractive bioinformatics approaches to the degradome sequence data to significantly reduce and simplify a metagenomic dataset for detection and assembly of complete genomes of plant viruses.

### SIMPLIFYING KNOWN SEQUENCE DATASETS

Segata et al. (2012) have explored a strategy (MetaPhlAn) in which sets of marker genes specific to species or higher level taxa are placed in a database that is only 4% the size of the nr database. Their search strategy maps the reads to this reduced set of sequences without the prior assembly of the reads. It yields abundances of known organisms and does not need prior filtering to remove errors and does not require annotation of reads. Reads can be assigned at 450/s. An alternative, Phymm, is to generate oligonucleotides characteristic of specific taxonomic groupings by interpolated Markov models. In a strategy similar to MetaPHlAn, but less rapid, Phymm can be coupled to BLAST (PhymmBL; Brady and Salzberg, 2011). A preclassification of database targets according to k-mer word contents enhances speed (available in USEARCH, Edgar, 2010) by preventing exhaustive further searching once a good hit has been found. As a result, in effect, each query searches a less than full database. A condensed database consisting of the taxonomically most informative 18 or 20 k-mers from the raw genome database and associated with their NCBI taxonomic identifiers has also been constructed and used to speed analysis in Livermore Metagenomics Analysis Toolkit (Ames et al., 2013), which uses k-mer matching as in Kraken.

Protein sequence databases can be substituted for the nucleotide sequence databases, in which case a BLAST search will utilize the BLASTx option (Zhao et al., 2012). Alternatively, databases of conserved protein sequences, such as Pfam, have been searched with translated queries using a Hidden Markov Model in software tools such as CARMA (Krause et al., 2008) and Treephyler (Schreiber et al., 2010). Alphabet reduction (Zhao et al., 2012; Huson and Xie, 2014) can further accelerate the amino acid sequence approaches. In these cases, non-coding sequences would be prevented from taking part in the taxonomic assignment of sequences and nucleotide variations, often important for finer taxonomic discriminations, are lost.

Whether protein or nucleotide sequence target databases are used, analysis of metagenomic datasets by BLAST search using NGS data as query is time consuming. With the large numbers of sequences currently being added to the databases, the prospects are for query times to lengthen rather than shorten for all. An additional problem for plant-based metagenomics is the likely presence of uncharacterized microbes of all types. Research on the human microbiome is aided by recent careful studies and characterization of pathogens and symbionts. There are virtually no data describing the microbiomes of plants in their many natural environments.

Martin et al. (2012) approached the problem by restricting their spectrum of organisms whose sequences were to be the targets of comparison. The whole genomes of the chosen targets for the human microbiome project were used as reference genomes against which six alignment programs mapped the reads (Martin et al., 2012). In another approach (Liu et al., 2011), the target sequence dataset was reduced considerably by focusing on a carefully chosen set of 31 marker genes that allow higher level taxonomic assignment. The reads were then mapped against these marker genes. The resulting Metaphyler software accomplished assignment in 8 h as opposed to 34 days for assignment using BLASTn and MEGAN.

### FLIPPING THE SEARCH

Concerns for plant biosecurity motivated the development of e-probe diagnostic nucleic acid analysis (EDNA; Stobbe et al., 2013). For plant biosecurity, it is important to know that particularly hazardous organisms are not present in materials imported across borders (Macdiarmid et al., 2013). For example, Race 3 biovar 2 of *Ralstonia solanacearum* is thought to have entered the United States on imported geranium plants (Kim et al., 2002). Plant biosecurity includes not only invasion of pathogens from abroad but also internal bioterrorist attacks. A prime defense against such bioterrorism is an excellent microbial forensics ability (Fletcher et al., 2010a,b). The microbial profiles of crime scene objects should clarify which objects are associated with the crime and should lead to comparisons with objects in a suspect’s hands (Smith, 2007). In plant biosecurity: the question asked is: which, if any, of a list of pathogenic organisms of concern are present.

EDNA simplifies answering these questions by presenting a complete reversal of the current standard procedure of operation. Instead of using the NGS sequences as queries of the ever-expanding general database, the NGS sequences are formatted to a BLAST searchable database, to be queried with panels of pretested probes specific for whatever taxonomic level is desired (Stobbe et al., 2013). By comparison of the target organism’s sequence with that of near relatives, a set of oligonucleotide sequences of a specified length is generated and tested for specificity against a general database. The surviving sequences are designated as “e-probes.” Such probes and their reversals, designated “decoy probes,” are used in BLASTn searches of unassembled, non-quality-checked metagenomic sequence reads formatted in a BLAST database. E-probes have been designed for a selected group of bacteria, viruses, fungi, and oomycetes. E-probe lengths of 80 or more nucleotides gave good discriminatory power. Statistical tests for comparing the results of e-probe searches with those of decoy-probe searches were devised to provide confidence levels in an identification of presence or absence of the target in the NGS dataset. EDNA analysis required no assembly or filtering, considered all portions of the NGS data (10–20 Mnt) and took only minutes to run on a typical laptop. EDNA was initially developed to aid in screening plant materials coming into quarantine for the presence or absence of pathogenic microorganisms of concern. It has applications also in phytopathological diagnostics. For example, metagenomes from three diseased plants were prepared and screened with plant virus electronic probes, resulting in the identification of a potexvirus in one of the samples and allowing further investigation of whether this virus had produced the disease (Stobbe, 2013).

EDNA is well suited to association–dissociation studies, and revealing endosymbionts and commensals. EDNA suffers from the requirement that the investigator needs to know, not only which organisms should be tested for, but also the nucleotide sequences of at least a large part of the genomes of those organisms, for the design of e-probes. However, it may be possible to design e-probe sets that recognize sequences specific at higher taxonomic levels than species. Such e-probe sets may lead to the recognition of previously unknown microbes, but only if they are related to known microbes. The design of e-probes that distinguish among viral strains has been demonstrated (Stobbe et al., 2014).

## CONCLUSION

The understanding of microbe–plant interactions will be improved by the knowledge of how multiple microbes interact with each other and with their hosts. NGS has the potential to generate such knowledge but requires computational improvement to accelerate the discovery process. The development of multiple strategies to produce such improvements portends adoption of NGS as a major tool for phytobiome exploration. The strategies include increasing computing speed, condensing the NGS sequence dataset, enriching for microbe sequence, simplifying known sequence datasets and changing the direction of BLAST searches. The latter, a property of the EDNA strategy, using e-probes in BLAST searches, has the potential of assisting investigation of interactions of multiple microbes with each other and the plant.

Clearly, dissection of the molecular details of multimicrobe interactions with plants will require experimentation on model systems with known combinations of microbes in green houses and growth chambers. On the other hand, knowing which multimicrobe–plant interactions are in need of investigation can best be facilitated by a metagenomic approach that correlates the presence of specific sets of microbes with physiological and developmental phenotypes in field-grown crops or in naturally growing non-cultivated stands of plants.

## AUTHOR CONTRIBUTIONS

Ulrich Melcher provided the concept for the article and created a draft; Ruchi Verma and William L. Schneider contributed improvements to the draft; William L. Schneider is the originator of the EDNA concept discussed in this article. All authors have contributed to the revision and editing of the article and approved its submission.

## Conflict of Interest Statement

The authors declare that the research was conducted in the absence of any commercial or financial relationships that could be construed as a potential conflict of interest.
